# Development of and Access to Products for Neglected Diseases

**DOI:** 10.1371/journal.pone.0010610

**Published:** 2010-05-12

**Authors:** Joshua Cohen, Maria Staroselsky Dibner, Andrew Wilson

**Affiliations:** 1 Tufts University Center for the Study of Drug Development, Boston, Massachusetts, United States of America; 2 Lahey Clinic, Department of Medicine, Burlington, Massachusetts, United States of America; Mount Sinai School of Medicine, United States of America

## Abstract

**Introduction:**

Prior research on neglected disease drug development suggested inadequate funding was responsible for relatively few new approvals. In response, significantly more resources have been allocated towards development of drugs targeting neglected diseases. Our objective was to reassess drug development between1975 and 1999, evaluate progress in neglected disease drug development since 2000, and explain how increased numbers of approvals are a necessary but insufficient condition to improving access.

**Methods:**

To assess numbers of approvals targeting neglected diseases, we employed two distinct methodologies: First, to revisit numbers published in Trouiller et al. (2002) we used their method to count marketed new chemical entities (NCEs) between 1975 and 1999. Second, using the G-Finder report as a benchmark, we identified which diseases are currently considered “neglected” to tally approvals in the 1975–1999 and 2000–2009 periods. Searching PharmaProjects and IMS R&D Focus databases as well as websites from numerous drug regulatory agencies, we identified new drug approvals and indications. Also, we examined the World Health Organization's (WHO) Essential Drug List (EDL) to see which drugs and indications were on the list.

**Findings:**

Upon recount, using Trouiller et al. methodology, we found that between 1975 and 1999 more NCEs (n = 32) targeting tropical diseases and tuberculosis were approved than reported in Trouiller et al. (n = 16). Using the G-Finder method of defining neglected diseases, we found 46 new drug approvals between 1975 and 1999. WHO included 85% of these drugs on the EDL. In the period 2000 to May 2009, despite much greater funding, only 26 new drugs and vaccines for neglected diseases were marketed. Of these, WHO placed 50% on the EDL.

**Conclusions:**

Product approvals for neglected diseases have increased, though progress has been uneven, with malaria appearing to benefit most in the short run from increased funding, while less success has been booked in other disease categories. Uneven progress suggests funding could be better targeted, particularly with regard to neglected diseases that have hitherto received scant attention. In addition, policymakers should focus on other aspects related to access. Besides drug development, there are the issues of EDL listing, architecture, availability, affordability, and adoption.

## Introduction

Neglected diseases are infectious diseases that primarily, though not exclusively, affect vulnerable populations in developing countries where poor sanitation and lack of access to health care foster disease transmission and vector proliferation. These diseases, which include malaria, tuberculosis, diarrheal diseases, and kinetoplastids such as leishmaniasis, cause 35,000 deaths per day in the developing world along with significant morbidity.[1]

There is great interest in the public health community in developing new products to treat or prevent these diseases. However, given limited health care budgets in most developing nations, the general public's weak purchasing power, and the correspondingly low likelihood of a satisfactory return on investment, there is comparatively little incentive for private industry to dedicate research and development (R&D) resources to developing medicines for these markets.[2]

In a widely cited 2002 study, Trouiller et al. reported that of 1393 new chemical entities (NCEs) marketed between 1975 and 1999, only 16 targeted “tropical diseases” and tuberculosis.[3] Furthermore, Trouiller et al. found that in 1999 less than $70 million was invested in drug research and development for malaria, tuberculosis, leishmaniasis, and African trypanosomias combined.[4]Their study galvanized thought leaders to proclaim the necessity of greater investment in neglected disease drug development. As such it served as a clarion call to action for governments, non-profit foundations, private-public partnerships, and the private industry to earmark more resources to battle this public health problem.

Since 1999, funding has greatly increased.[5] Additionally, definitions of neglected disease have expanded beyond the tropical diseases and tuberculosis included in Trouiller et al. Large new cooperative ventures have begun to take shape, including the Global Network for Neglected Tropical Disease Control, Medicines for Malaria Venture (MMV), the Drugs for Neglected Diseases Initiative (DNDi), and numerous partnerships with pharmaceutical companies, including Merck, GlaxoSmithKline, Pfizer, Novartis and Sanofi-Aventis. According to the G-Finder report, in 2007, $2.5 billion was invested in neglected disease drug development, with $980 million targeting malaria, tuberculosis, and the kinetoplastids combined. Nearly 80% of funds poured into the “big three:” HIV/AIDS, tuberculosis, and malaria. Of this amount, nearly 90% came from public or philanthropic donors, with the National Institutes of Health and the Bill and Melinda Gates Foundation leading the way.[6]

In comparison with a decade ago, more resources are being spent to address the problem of neglected diseases. A way of addressing the question of whether increased funding has been effective is to mark progress in new approvals. A progress report was published in 2006, which showed several new approvals over the 2000–2004 period.[7] However, our study provides an updated, in-depth examination of new approvals through May of 2009. We also analyze whether new approvals are being included in the WHO's EDL. Together with WHO treatment guidelines, the EDL forms the basis for public health policy in many developing countries. Finally, we examine the larger question of pharmaceutical access.

First, our paper revisits numbers of approved drugs targeting “tropical diseases” and tuberculosis previously published by Trouiller et al. Second, we mark progress in neglected disease drug approvals since 1999. Finally, we explain how increased numbers of approvals are a necessary but insufficient condition to improving access.

## Methods

To assess new approvals targeting neglected diseases, we employed two distinct methodologies: First, we used the method in Trouiller et al. to count marketed new chemical entities (NCEs) between 1975 and 1999, as well as fixed dose combination products. Second, referencing the authoritative G-Finder report as a benchmark, we identified currently defined neglected diseases to tally approved products and indications in the 1975–1999 and 2000–2009 periods. The G-Finder report investigated funding allocated to the following diseases: malaria, tuberculosis, bacterial pneumonia and meningitis, pneumonia, rotavirus, enterotoxigenic and enteroaggregative E. Coli, cholera, shigella, cryptosporidium, giardia, Chagas disease, leishmaniasis, African trypanosomiasis, roundworm (ascariasis), hookworm (ancylostomiasis & necatoriasis), whipworm (trichuriasis), strongyloidiasis and other intestinal roundworms, lymphatic filariasis (elephantiasis), onchocerciasis (river blindness), schistosomiasis (bilharziasis), tapeworm (cystercercosis/taeniasis), leprosy, trachoma, Buruli ulcer, Dengue fever, rheumatic fever, typhoid and paratyphoid fever, and HIV/AIDS (products with applications specific to the developing world, such as vaccines, microbicides and pediatric label extensions). This enumeration of diseases builds upon Trouiller et al. Besides the inclusion of Buruli ulcer, bacterial pneumonia and meningitis, rheumatic fever, typhoid and paratyphoid fever, and the exclusion of Japanese encephalitis, the biggest difference is that HIV/AIDS drugs and anti-diarrhoeals are included in the G-Finder count, so long as they are indicated for conditions pertinent to the developing world. In the case of HIV/AIDS products this implies pediatric use, vaccines and microbicides. Anti-diarrhoeal drugs were only counted if indicated for cholera, shigella, or cryptosporidium. Finally, anti-diarrhoeal vaccines were included if they targeted one or more diseases across the entire diarrhoeal spectrum.

In contrast to Trouiller et al., we categorized indications separately in our G-Finder based count. If a drug received approval for more than one disease, we counted each instance as a newly approved indication though only one instance as a newly approved drug. For example, mebendazole can be used for four different helminths, so it was counted for four indications, but just once as a newly approved drug. Note that new doses or formulations of (combination) drugs did not count towards new approvals.

We identified new product approvals and indications using the PharmaProjects and IMS R&D Focus databases, websites from the Food and Drug Administration, European Medicines Agency, and drug regulatory agencies of France, Germany, the Netherlands, the United Kingdom, China, Kenya, and India. We also examined the most recent version of the EDL to see which drugs, indications, and vaccines are recommended for use.[8]

## Results

Using the same NCE count methodology employed in Trouiller et al. we found that their figure of 16 appears to have undercounted the total number of drugs approved for “tropical diseases” and tuberculosis between 1975 and 1999; namely, 32. Moreover, the Trouiller et al. list is inaccurate as five of the 16 drugs were not approved between 1975 and 1999.

According to our tally based on a G-Finder definition of neglected diseases, 46 new products were approved between 1975 and 1999 targeting neglected diseases, with a total of 56 indications. Of these, 6 were for pediatric HIV (note, no microbicides or vaccines were approved), 7 for malaria, 12 for tuberculosis, three for bacterial pneumonia and meningitis, two new drugs and four new indications for diarrheal diseases, two for kinetoplastids, 9 new drugs and 16 new indications for helminths, two for leprosy and one each for trachoma, rheumatic fever, and typhoid fever. No new products were approved for Buruli ulcer and Dengue fever. Of the 46 new drug approvals, 39 (85%) were placed on the EDL. And, of the 56 new indications approved for marketing, 46 (82%) were added to the EDL.

In table 1 we list the original Trouiller et al. numbers, our recount of Trouiller et al., an analysis of numbers based on a broader G-Finder definition of neglected diseases, and a tally of percentages of drugs and indications on the EDL. The appendix (Appendix S1) includes tables which provide a detailed count of all approvals, their year of approval, and the regulatory authority that made the initial approval.

**Table 1:** Numbers of 1975-1999 Approvals for Neglected Diseases.

Disease Category	Trouiller et al. new chemical entities (NCEs) targeting “tropical diseases” and tuberculosis	Our Analysis		
		Our recount of Trouiller et al. NCEs	Our tally of G-Finder defined drugs targeting neglected diseases	Our tally of G-Finder defined indications targeting neglected diseases
HIV/AIDS*	n/a**	n/a**	6***	6
Malaria	4	7	7	7
Tuberculosis	3	12	12	12
Bacterial Pneumonia and Meningitis	n/a**	n/a**	3	3
Diarrheal Diseases	n/a**	n/a**	2	4
Kinetoplastids	5	2	2	2
Buruli Ulcer	n/a**	0	0	1
Dengue Fever	0	0	0	0
Helminths	4	9	9	16
Leprosy	0	2	2	2
Trachoma	n/a**	n/a**	1	1
Rheumatic Fever	n/a**	n/a**	1	1
Typhoid and Paratyphoid Fever	n/a**	n/a**	1	1
Total Approvals	16	32	46	56
Percentage on Essential Drug List	94%	85%	85%	82%

*In their analysis, Trouiller et al. included 26 HIV drugs-20 anti-virals and 6 drugs for “opportunistic diseases”-but as a separate (non-neglected) disease category.

**Disease categories Trouiller et al. did not include in their analysis.

***HIV/AIDS drugs with applications specific to the developing world, such as vaccines, microbicides and pediatric label extensions. Sources: PharmaProjects, IMS R&D Focus, http://www.accessdata.fda.gov/Scripts/cder/DrugsatFDA, http://eudrapharm.eu/eudrapharm/searchbykeyword.do, http://www.cbg-meb.nl/CBG/en/human-medicines/actueel, http://www.pharmacyboardkenya.org/index.php?id=13&dpgndg1=42&an=, http://www.who.int/medicines/publications/08_ENGLISH_indexFINAL_EML15.pdf, http://www.cdsco.nic.in/, http://www.afssaps.fr/, http://www.mhra.gov.uk/index.htm, http://www.bmg.bund.de.

Between 2000 and May 2009, 26 products for neglected diseases were marketed with a total of 26 indications. Of these, the WHO had placed 50% on the 2009 EDL. The greatest number of approvals occurred in malaria with 11 new drugs being marketed. An additional 10 new HIV/AIDS drugs were granted pediatric labeling; one new drug and two vaccines for diarrheal diseases; one vaccine was developed against bacterial meningitis, and one new drug was approved for kinetoplastids. No other disease category had any new drugs approved in the last 9 years. Table 2 lists all products approved for neglected diseases between 2000 and May 2009 along with their EDL status.

**Table 2:** Numbers of 2000–2009 Approvals for G-Finder Defined Neglected Diseases.

Disease Category	Our Analysis	
	Drugs/Vaccines	Indications
HIV/AIDS*	10	10
Malaria	11	11
Tuberculosis	0	0
Bacterial Pneumonia and Meningitis	1	1
Diarrheal Diseases	3	3
Kinetoplastids	1	1
Buruli Ulcer	0	0
Dengue Fever	0	0
Helminths	0	0
Leprosy	0	0
Trachoma	0	0
Rheumatic Fever	0	0
Typhoid and Paratyphoid Fever	0	0
Total Approvals	26	26
Percentage on Essential Drug List	50%	50%

*HIV/AIDS drugs with applications specific to the developing world, such as vaccines, microbicides and pediatric label extensions. Sources: PharmaProjects, IMS R&D Focus, http://www.accessdata.fda.gov/Scripts/cder/DrugsatFDA, http://eudrapharm.eu/eudrapharm/searchbykeyword.do, http://www.cbg-meb.nl/CBG/en/human-medicines/actueel, http://www.pharmacyboardkenya.org/index.php?id=13&dpgndg1=42&an=, http://www.who.int/medicines/publications/08_ENGLISH_indexFINAL_EML15.pdf, http://www.cdsco.nic.in/, http://www.afssaps.fr/, http://www.mhra.gov.uk/index.htm, http://www.bmg.bund.de.

For a complete listing of all products and indications approved in the 1975–1999 and 2000–2009 periods, please see appendix (Appendix S1).

The percentage of approved neglected disease products sponsored by the private pharmaceutical industry dropped from 83% to 46% between the two time periods, while the percentage sponsored by public-private partnerships increased from 15% to 46% (see table 3). Here, sponsorship implies the sponsor paid for a product's development through the clinical phases.

**Table 3:** Comparison of Approvals by Source of Sponsorship.

	1975–1999	2000–2009
Number of Individual Products Approved	46	26
Number of Products Sponsored by PPP (% of Total)	7 (15%)	12 (46%)
Number Sponsored by Private Industry (%)	38 (83%)	12 (46%)
Number Sponsored by Other/Unknown (%)	1 (2%)	2 (8%)

Sources: PharmaProjects, IMS R&D Focus, http://www.accessdata.fda.gov/Scripts/cder/DrugsatFDA, http://eudrapharm.eu/eudrapharm/searchbykeyword.do, http://www.cbg-meb.nl/CBG/en/human-medicines/actueel, http://www.pharmacyboardkenya.org/index.php?id=13&dpgndg1=42&an=, http://www.who.int/medicines/publications/08_ENGLISH_indexFINAL_EML15.pdf, http://www.cdsco.nic.in/, http://www.afssaps.fr/, http://www.mhra.gov.uk/index.htm, http://www.bmg.bund.de.

It may be problematic to mark progress using numbers of approvals. Clearly, many of the product development efforts that began in 2000–2009 have not (yet) resulted in new product approvals, given the (variable) length of time between initial funding of R&D and registration. Indeed, collecting data on new products in clinical development, we see promising signs as 97 are currently in the pipeline despite the fact that certain therapeutic areas continue to be neglected (see table 4). This said, we do not think it is far-fetched to already be looking for indications of progress in terms of numbers of new approvals, particularly given the fact that the conventional 10–15 year period to register a drug may not invariably apply as numerous products currently being developed by PPPs have been granted accelerated approval times.[9]

**Table 4:** Neglected Disease Products in Clinical Development as of July 2009.

Disease Category	Drugs	Vaccines	Microbicides
HIV/AIDS	0	24	4
Malaria	9	19	—
Tuberculosis	5	7	—
Bacterial Pneumonia and Meningitis	0	4	—
Diarrheal Diseases	0	11	—
Kineptoplastids	4	2	—
Buruli Ulcer	0	0	—
Dengue Fever	0	3	—
Helminths	1	1	—
Leprosy	0	0	—
Trachoma	0	0	—
Rheumatic Fever	0	0	—
Typhoid and Paratyphoid Fever	0	3	—
Total	19	74	4

Sources: BIO Ventures for Global-R&D Landscape, <http://www.bvgh.org/resources/landscape/default.asp>; Cowen and Co. Pharmaceuticals Industry Overview, May 2009; PhRMA Medicines in Development Factsheets for HIV/AIDS, infectious disease, and biotechnology; International Federation of Pharmaceutical Manufacturers and Associations, “Pharmaceutical Industry R&D for Diseases of the Developing World-2009”, <http://www.ifpma.org/documents/NR12400/Status_RnD_for_DDW_07Jul09.pdf>; ClinicalTrials.gov; Moran, et al. (2007) “The Malaria Product Pipeline: Planning for the Future.” The George Institute for International Health, <http://www.thegeorgeinstitute.org/research/health-policy/current-projects/the-malaria-product-pipeline-planning-for-the-future.cfm>; websites, press releases, and reports from various PDPs and NGOs including the Meningitis Vaccine Project, PATH (including Malaria Vaccine Initative), Medicines for Malaria Venture, Alliance for Microbicide Development, Drugs for Neglected Diseases Initiative, International AIDS Vaccine Initiative, AIDS Vaccine Advocacy Coalition, Global Alliance for TB Drug Development Note to table 4: Table 4 presents a snapshot of active drugs, vaccines, and microbicides in clinical development for neglected diseases as of July 2009. Using the inclusion criteria outlined in Moran et al., the majority of products were located using commercial and public reports from trade associations (PhRMA, IFPMA), commercial entities (Cowen and Co.), various product development partnerships (PDPs) and NGOs (BioVentures for Global Health), and government agencies such as the NIH and US Department of Defense. Products were only included when their status could be verified through secondary sources, which included clinical trial records from ClinicalTrials.gov, information posted on company websites, and website information from the sources listed in the table. The table should not be considered an exhaustive list of every product in development. First, only new products were listed, which excludes previously approved products that are now being developed for wider use. For example, the dihydroartemisinin + piperaquine combination currently in pre-registration was left out because it was approved in Kenya in 2005 (see table 2), despite being considered by some analysts as part of the pipeline. Second, our reliance on public and commercial sources potentially overlooks other sources.

## Discussion

Regardless of how one does the counting, the 1975–1999 period was not good for neglected disease product development. However, since Trouiller's call to action, there has been progress in neglected disease product development, albeit in uneven strides. For example, malaria has seen a 250% increase in numbers of new products compared to the 1975–1999 period. Nevertheless, malaria appears to be the exception rather than the rule. While tuberculosis has received similar funding to malaria, not a single new tuberculosis drug has been approved in the last nine years. Although this may be in part due to longer development times for tuberculosis products, we also observe fewer products in the clinical development pipeline than with malaria. Likewise, despite HIV/AIDS R&D towards applications specific to the developing world totaling $1.8 billion in 2007, of which 64% went to vaccine development and 18% to microbicides, no vaccines or microbicides have been approved. Finally, not a single new product has been approved in the last 9 years in disease categories that include Buruli ulcer, Dengue fever, trachoma, rheumatic fever, or typhoid and paratyphoid fevers.

Here, we do not wish to leave the impression that the now predominant PPP model is not promising. Besides having numerous products in the clinical development pipeline that may prove invaluable, PPPs have demonstrated an ability to develop drugs with high health impact. PPP-based products, such as the arthemeter + lumefantrine combination, are making a difference in the developing world.

Separate from R&D funding levels and new approvals is the issue of inclusion on the EDL, which does not guarantee access but may act as a lever to increase access. The EDL recommends what it considers to be the most essential, yet cost-effective drugs available. As such, it forms the basis for public health policy in many developing nations.[10] There are clear linkages between EDL, clinical practice guidelines, and drug procurement practices. These relationships underscore the “operational, educational, and symbolic functions of an essential drugs list:”[11] Operational in that the EDL identifies medicines for priority attention for acquisition and distribution; educational in that the EDL influences drug utilization patterns; and symbolic in that the EDL confers global recognition and a preferred position in pharmaceutical management.

Of the drugs targeting neglected diseases that were developed between 1975 and 1999, 85% are on the EDL. By comparison, only 50% of products approved in the 2000–2009 period are on the EDL. This could be due to lag time as the WHO deliberates following each new approval, but may also be the result of the higher cost of certain newer drugs.[12] In a severely budget-constrained environment the low cost of older drugs may tip the scales in their favor. Needless to say, non-admittance to the EDL can serve as an access barrier, with possibly deleterious health outcomes.

It is often assumed that once a product is added to the EDL, the access problem is resolved. However, besides EDL listing, numerous barriers to access persist, the foremost being resource constraints. Others include limited capacity of public health systems, lack of political commitment in terms of distribution and delivery of products and services, international trade and patent disputes, and cultural attitudes towards disease and products to remedy or prevent disease.[13]

Access also comprises availability, affordability, adoption, and architecture. Availability refers to “the logistics of making, ordering, shipping, storing, distributing and delivering a new health technology to ensure it reaches the hands (or mouth) of the user.”[14] This implies that even if a product travels the drug development route successfully, reaches market, and is recommended by WHO for use, there is still the question of how it is to be distributed to the people who need it. Affordability encompasses both the individual patient's ability to pay and that of governments and other payers. This is less of a concern for medications such as ivermectin, which Merck donates. But, it may be an acute issue for drugs such as artemisisin-based combination therapies.[15] Adoption runs the gamut from a product's recommendation by international agencies like WHO to its acceptance by local policymakers in developing countries, to patients as well as health care providers, some of whom may have misgivings about taking certain products. Lastly, architecture refers to the organizational dimension of access; decisions about organizational structure that are required for coordinating availability, affordability, and adoption. In this context, consider, for example, the anti-helminths. Given that there are already 9 drugs that are effective at treating helminths, it appears that the need for development of new anti-helminths is less critical than the need for improved access. Hence, the helminths appear to be neglected diseases not because of a drug deficit but due to limited effective means of getting these drugs to the people who need them.

In sum, funding of neglected disease R&D is highly concentrated, with significant funding flowing into HIV/AIDS, malaria and tuberculosis product development. Progress is lopsided, with marked strides in the area of malaria research, yet few end products in others. This suggests the infusion of more money itself is insufficient, while better targeting of funds may be warranted. Moreover, a balanced comprehensive approach to address the neglected disease problem will involve not only drug development but also attention paid to public health infrastructure and capacity-building to improve access.

## Supporting Information

Appendix S1 *Trouiller et al. did not include year of approval for each drug, nor the regulatory agency that made the initial approval. Using multiple sources, including IMS R&D Focus and PharmaProjects, we identified both year of approval and the regulatory agency that made the initial approval. **Trouiller et al. included fivedrugs on their 1975–1999 approvals list that were not approved during that period. First, they included pyrazinamide as a separate NCE. Our research found that this drug was first approved in 1954. However, we did find that pyrazinamide was approved as part of a combination product in 1998 (isoniazid +rifampicin + pyrazinamide + ethambutol). Second, Trouiller et al. included the atovaquone + proguanil combination as a 1975–1999 approval. However, this combination product was approved in 2000. Therefore, in our list we included it as a 2000–2008 approval. Third, Trouiller et al. included benznidazole as a drug approved between 1975 and 1999. Our research found that benznidazole was first approved in 1972. Therefore, we did not include it on our list of 1975–1999 approvals. Fourth, Trouiller et al. included nifurtimox as a drug approved between 1975 and 1999. Our research found that nifurtimox was first launched in 1967 as a drug targeting Chagas' disease. Therefore, we did not include it on our list of 1975–1999 approvals. Fifth, Trouiller et al. included pentamidine in the 1975–1999 approvals list. Our research found that pentamidine was first approved in France in 1956 as a kinetoplastid.
(0.14 MB DOC)
