# Supplementary material for: Development of and Access to Products for Neglected Diseases
Source: PLoS One. 2010 May 12;5(5):e10610. doi: 10.1371/journal.pone.0010610 (PMC2868904; doi:10.1371/journal.pone.0010610)
Supplement: Appendix S1 — *Trouiller et al. did not include year of approval for each drug, nor the regulatory agency that made the initial approval. Using multiple sources, including IMS R&D Focus and PharmaProjects, we identified both year of approval and the regulatory agency that made the initial approval. **Trouiller et al. included fivedrugs on their 1975–1999 approvals list that were not approved during that period. First, they included pyrazinamide as a separate NCE. Our research found that this drug was first approved in 1954. However, we did find that pyrazinamide was approved as part of a combination product in 1998 (isoniazid +rifampicin + pyrazinamide + ethambutol). Second, Trouiller et al. included the atovaquone + proguanil combination as a 1975–1999 approval. However, this combination product was approved in 2000. Therefore, in our list we included it as a 2000–2008 approval. Third, Trouiller et al. included benznidazole as a drug approved between 1975 and 1999. Our research found that benznidazole was first approved in 1972. Therefore, we did not include it on our list of 1975–1999 approvals. Fourth, Trouiller et al. included nifurtimox as a drug approved between 1975 and 1999. Our research found that nifurtimox was first launched in 1967 as a drug targeting Chagas' disease. Therefore, we did not include it on our list of 1975–1999 approvals. Fifth, Trouiller et al. included pentamidine in the 1975–1999 approvals list. Our research found that pentamidine was first approved in France in 1956 as a kinetoplastid. (0.14 MB DOC) [file pone.0010610.s001.doc]

Appendix

Table A

Trouiller et al. approvals for “tropical diseases” and tuberculosis, 1975-1999

| **Disease** | **Drug** | **Year of Approval*** | **Drug Regulatory Agency** |
| --- | --- | --- | --- |
| *Tuberculosis* | Pyrazinamide** | 1954 | France |
|  | Rifabutin | 1992 | FDA |
|  | Rifapentine | 1998 | FDA |
|  |  |  |  |
|  |  |  |  |
| *Malaria* | Arthemether | 1990 | China |
|  | Mefloquine | 1992 | FDA |
|  | Halofantrine | 1992 | FDA |
|  | Atovaquone + Proguanil** | 2000 | FDA |
|  |  |  |  |
| *Tropical Diseases* | Benznidazole** | 1972 | FDA |
|  | Nifurtimox** | 1967 | Argentina |
|  | Albendazole | 1981 | France |
|  | Eflornithine | 1990 | FDA |
|  | Oxamniquine | 1980 | FDA |
|  | Ivermectin | 1996 | FDA |
|  | Liposomal amphotericin B | 1991 | UK |
|  | Praziquantel | 1981 | Germany |
|  | Pentamidine** | 1952 | France |

*Trouiller et al. did not include year of approval for each drug, nor the regulatory agency that made the initial approval. Using multiple sources, including IMS R&D Focus and PharmaProjects, we identified both year of approval and the regulatory agency that made the initial approval.

**Trouiller et al. included fivedrugs on their 1975-1999 approvals list that were not approved during that period. First, they included pyrazinamide as a separate NCE. Our research found that this drug was first approved in 1954. However, we did find that pyrazinamide was approved as part of a combination product in 1998 (isoniazid + rifampicin + pyrazinamide + ethambutol). Second, Trouiller et al. included the atovaquone + proguanil combination as a 1975-1999 approval. However, this combination product was approved in 2000. Therefore, in our list we included it as a 2000-2008 approval. Third, Trouiller et al. included benznidazole as a drug approved between 1975 and 1999. Our research found that benznidazole was first approved in 1972. Therefore, we did not include it on our list of 1975-1999 approvals. Fourth, Trouiller et al. included nifurtimox as a drug approved between 1975 and 1999. Our research found that nifurtimox was first launched in 1967 as a drug targeting Chagas’ disease. Therefore, we did not include it on our list of 1975-1999 approvals. Fifth, Trouiller et al. included pentamidine in the 1975-1999 approvals list. Our research found that pentamidine was first approved in France in 1956 as a kinetoplastid.

Table B

Our list of approvals for G-Finder neglected diseases, 1975-1999

| **Disease** | **Drug** | **Year of Approval** | **Drug Regulatory Agency** |
| --- | --- | --- | --- |
| *HIV/AIDS* | Zidovudine ZDV + Azidothymidine AZT | 1990 | FDA |
|  | Didanosine + Dideoxynosine DDL | 1999 | FDA |
|  | Abacavir ABC | 1998 | FDA |
|  | Efavirenz EFV | 1998 | FDA |
|  | Nevirapine NVP | 1996 | FDA |
|  | Amprenavir APV | 1999 | FDA |
|  |  |  |  |
|  |  |  |  |
|  |  |  |  |
| *Malaria* | Halofantrine | 1992 | FDA |
|  | Amodiaquine | 1998 | Kenya |
|  | Mefloquine | 1992 | FDA |
|  | Sulfadoxine + Pyrimethamine | 1981 | FDA |
|  | Artemisinin | 1996 | France |
|  | Arthemeter | 1990 | China |
|  | Arthemeter + Lumefantrine | 1999 | Kenya |
| *Tuberculosis* | Rifapentine | 1998 | FDA |
|  | Kanamycin | 1981 | FDA |
|  | Amikacin | 1981 | FDA |
|  | Ofloxacin | 1990 | FDA |
|  | Ciprofloxacin | 1987 | FDA |
|  | Ethambutol + Isoniazid | 1995 | Kenya |
|  | Isoniazid + Pyrazinamide + Rifabutin | 1999 | FDA |
|  | Isoniazid + Rifabutin | 1975 | FDA |
|  | Isoniazid + Rifampicin + Pyrazinamide + Ethambutol | 1998 | Kenya |
|  | Rifabutin | 1992 | FDA |
|  | Moxifloxacin | 1999 | FDA |
|  | Thiacetazone + Isoniazid | 1996 | Kenya |
|  |  |  |  |
| *Bacterial Pneumonia and Meningitis* | Ceftriaxone | 1984 | FDA |
|  | Cefotaxime | 1981 | FDA |
|  | Amoxicillin + Clavulanic acid | 1984 | FDA |
|  |  |  |  |
| *Diarrheal Diseases* |  |  |  |
|  | Ceftazidime | 1985 | FDA |
|  | Nalidixic acid | 1986 | FDA |
|  |  |  |  |
|  |  |  |  |
| *Kinetoplastids* | Liposomal amphotericin B | 1991 | UK |
|  | Eflornithine | 1990 | FDA |
|  |  |  |  |
| *Buruli Ulcer* | None |  |  |
|  |  |  |  |
| *Dengue Fever* | None |  |  |
|  |  |  |  |
| *Helminths* | Albendazole | 1981 | UK |
|  | Triclabendazole | 1997 | Egypt |
|  | Levamisole | 1990 | FDA |
|  | Mebendazole | 1995 | FDA |
|  | Pyrantel pamoate | 1988 | FDA |
|  | Ivermectin | 1996 | FDA |
|  | Oxamniquine | 1980 | FDA |
|  | Praziquantel | 1981 | Germany |
|  | Niclosamide | 1982 | FDA |
|  |  |  |  |
| *Leprosy* | Dapsone + Rifampicin | 1981 | Unknown* |
|  | Clofazimine + Rifampicin + Dapsone | 1981 | Unknown* |
|  |  |  |  |
| *Trachoma* | Azithromycin | 1991 | FDA |
|  |  |  |  |
| *Rheumatic Fever* | Sulfadiazine | 1992 | Australia |
|  |  |  |  |
| *Typhoid and paratyphoid fever* | Cefixime | 1989 | FDA |

Table C

Our list of approvals for G-Finder neglected diseases, 2000-2009

| **Disease** | **Drug** | **Year Approved** | **Country of Approval** |
| --- | --- | --- | --- |
| *HIV/AIDS* | Lamivudine + Zidovudine | 2002 | FDA |
|  | Lamivudine 3TC | 2002 | FDA |
|  | Stavudine D4T | 2002 | FDA |
|  | Ritonavir RTV | 2005 | FDA |
|  | Nelfinavir mesylate NPV | 2004 | FDA |
|  | Enfurvitide | 2006 | FDA |
|  | Efavirenz + Emtricitabine + Tenofovir | 2006 | FDA |
|  | Emtricitabine FTC | 2003 | FDA |
|  | Lopinavir + Ritonavir | 2000 | FDA |
|  | Fasamprenavir calcium | 2003 | FDA |
|  | Tipranavir |  |  |
| *Malaria* | Atovaquone + Proguanil | 2000 | FDA |
|  | Artemisin + Amodiaquine | 2008 | Kenya |
|  | Artemotil | 2000 | Netherlands |
|  | Artesunate | 2001 | Kenya |
|  | Dihydroartemisinin | 2001 | Kenya |
|  | Dihydroartemisinin + Piperaquine | 2005 | Kenya |
|  | Artesunate + Mefloquine | 2005 | Kenya |
|  | Artesunate + Amodiaquine | 2007 | Morocco |
|  | Artesunate + Sulfadoxine + Pyrimethamine | 2007 | Kenya |
|  | Dihydroartemisinin + Piperaquine + Trimethoprim | 2006 | Kenya |
|  | Chlorproguanil + Dapsone | 2003 | Kenya |
|  |  |  |  |
| *Tuberculosis* | None |  |  |
|  |  |  |  |
| *Bacterial Pneumonia and Meningitis* | Menactra (polysaccharide diphtheria toxoid conjugate vaccine) | 2005 | FDA |
|  |  |  |  |
| *Diarrheal Diseases* | Pentavalent rotavirus vaccine | 2006 | FDA |
|  | Dukoral (diarrhea and cholera vaccine) | 2004 | UK |
|  | Nitazoxanide | 2004 | FDA |
|  |  |  |  |
| *Kinetoplastids* | Miltefosine | 2002 | India |
|  |  |  |  |
| *Buruli Ulcer* | None |  |  |
| *Dengue Fever* | None |  |  |
| *Helminths* | None |  |  |
| *Leprosy* | None |  |  |
| *Trachoma* | None |  |  |
| *Rheumatic Fever* | None |  |  |
| *Typhoid and paratyphoid fever* | None |  |  |
